# Registro Brasileiro de Cardiologia Intervencionista durante a Pandemia de COVID-19 (RBCI-COVID19)

**DOI:** 10.36660/abc.20220840

**Published:** 2023-08-10

**Authors:** Viviana Guzzo Lemke, Maria Sanali Souza Paiva, Giordana Zeferino Mariano, Thales Siqueira Alves, Esmeralci Ferreira, Leonardo Avany Nunes, Flavio Roberto Azevedo Oliveira, Rodrigo Cantarelli, Emilia Matos do Nascimento, Gláucia Maria Moraes de Oliveira

**Affiliations:** 1 Cardiocare Curitiba PR Brasil Cardiocare, Curitiba, PR – Brasil; 2 Hospital Universitário Onofre Lopes Natal RN Brasil Hospital Universitário Onofre Lopes – HUOL, Natal, RN – Brasil; 3 Hospital São João Batista Criciúma SC Brasil Hospital São João Batista, Criciúma, SC – Brasil; 4 Hospital Universitário Pedro Ernesto Rio de Janeiro RJ Brasil Hospital Universitário Pedro Ernesto, Rio de Janeiro, RJ – Brasil; 5 Universidade do Estado do Rio de Janeiro Rio de Janeiro RJ Brasil Universidade do Estado do Rio de Janeiro – Doenças do Tórax, Rio de Janeiro, RJ – Brasil; 6 Hospital Dom Helder Câmara Recife PE Brasil Hospital Dom Helder Câmara, Recife, PE – Brasil; 7 Universidade do Estado do Rio de Janeiro Rio de Janeito RJ Brasil Universidade do Estado do Rio de Janeiro, Rio de Janeito, RJ – Brasil; 8 Universidade Federal do Rio de Janeiro Rio de Janeiro RJ Brasil Universidade Federal do Rio de Janeiro, Rio de Janeiro, RJ – Brasil

**Keywords:** COVID-19, Myocardial Infarction, Percutaneous Coronary Intervention, Coronary Angiography, Coronary Artery Disease

## Abstract

**Fundamento:**

No início da pandemia de COVID-19, os pacientes com infarto do miocárdio (IM) demoraram para procurar um hospital por medo de contágio ou dificuldades no acesso aos serviços de saúde.

**Objetivos:**

Avaliar procedimentos de cardiologia intervencionista realizados durante a pandemia de COVID-19 e implicações na abordagem do IM.

**Métodos:**

Registro prospectivo de 24 centros de hemodinâmica no Brasil, com pacientes adultos submetidos a procedimentos de cardiologia intervencionista entre 26 de maio e 30 de novembro de 2020. Os desfechos foram complicações cardiovasculares (CV) e não CV, morte e IM. A concomitância de COVID-19 foi confirmada com RT-PCR. Técnicas de *machine learning* foram usadas com modelos não paramétricos de árvores de classificação. Usou-se análise de correspondência simples com o software R. Adotou-se nível de significância de 5%.

**Resultados:**

Este estudo incluiu 1.282 pacientes, 435 dos quais (33,9%) apresentaram IM: IM com supra de ST (IMCSST), 239 (54,9%); e IM sem supra de ST(IMSSST), 196 (45.1%). Dos 1.282 pacientes, 29 tiveram complicações CV, 47 tiveram complicações não CV e 31 morreram. O diagnóstico de COVID-19 foi confirmado em 77 pacientes (6%), com 15,58% de mortalidade e 6,49% de complicações não CV. A maioria dos pacientes apresentou significativa doença arterial coronariana (63%). Trombo intracoronariano foi mais frequente na presença de IMCSST (3,4%) e COVID-19 (4%). Tempo porta-mesa superior a 12 horas no IMSSST associou-se a 30,8% de complicações, 25% em pacientes com COVID-19.

**Conclusões:**

Todos os óbitos foram precedidos por complicações CV ou não CV. A presença de COVID-19 foi associada a óbito e complicações não fatais dos pacientes submetidos a procedimentos de cardiologia intervencionista durante a pandemia.

## Introdução

Até o início de 2022, cerca de 23 milhões de casos de COVID-19 e 621 mil mortes pela doença haviam sido registrados no Brasil.^1^ Durante 2020, a pandemia afastou os pacientes do tratamento de suas doenças cardiovasculares (DCV), especialmente as agudas, como infarto do miocárdio (IM) e acidente vascular cerebral (AVC), com consequente declínio e/ou atraso nas admissões hospitalares, pois os hospitais eram considerados locais perigosos devido ao risco de infecção.^2 - 4^

Estudo realizado no Brasil, utilizando o banco de dados público do Registro Civil, avaliou as mortalidades total e por excesso cardiovascular (CV) nas semanas 12 a 22 de 2020, nas seis cidades brasileiras com o maior número de mortes por COVID-19 (São Paulo, Rio de Janeiro, Fortaleza, Recife, Belém, Manaus) e comparou-as com as do mesmo período de 2019. Houve uma redução no excesso de mortes CV especificadas (IM e AVC) em paralelo com um aumento das mortes CV não especificadas e em domicílio.^5^

Vários estudos relataram mortes e significativas complicações cardíacas, como IM, eventos trombóticos, insuficiência cardíaca, miopericardite e arritmias cardíacas, em pacientes com COVID-19 com ou sem DCV prévia.^2 - 6^ O dano da COVID-19 ao sistema CV é provavelmente multifatorial e pode resultar, no início, de um desequilíbrio entre alta demanda metabólica e baixa reserva cardíaca associado com inflamação sistêmica e trombogênese, além de lesão cardíaca direta pelo SARS-CoV-2.^7 , 8^

Poucos estudos avaliaram o impacto da pandemia nos procedimentos de cardiologia intervencionista, assim como as implicações da pandemia na abordagem invasiva do IM na população brasileira.^9 , 10^ Portanto, este estudo teve por objetivo analisar os procedimentos percutâneos CV realizados durante a pandemia de COVID-19, assim como suas implicações para a abordagem invasiva do IM, usando um registro multicêntrico de laboratórios de hemodinâmica no Brasil (RBCI-COVID19).

## Métodos

Trata-se de registro multicêntrico observacional com dados de todos os pacientes consecutivos com idade mínima de 18 anos, ambos os sexos, submetidos a procedimento CV em 24 laboratórios de hemodinâmica no Brasil de 26 de maio a 30 de novembro de 2020 (Material Suplementar 1). Os critérios de exclusão foram: recusa em assinar o termo de consentimento, admissão para procedimentos neurológicos ou vasculares periféricos, ou implante de dispositivos para estimulação cardíaca artificial.

Os centros participantes foram incluídos por demanda espontânea após divulgação do registro em laboratórios de hemodinâmica de todas as regiões do Brasil. Os dados foram coletados a partir de fichas eletrônicas padronizadas, disponibilizadas *online* , e inseridos pelas equipes dos centros devidamente treinadas pelo centro coordenador.^11^

Os desfechos observados foram óbito por qualquer causa, IM e complicações CV (tamponamento cardíaco, parada cardiorrespiratória, nova revascularização, AVC, embolia pulmonar, insuficiência cardíaca aguda, choque cardiogênico) e não CV (infecção respiratória, sepse, choque séptico, hemorragia procedural). As complicações que ocorreram desde a chegada ao laboratório de hemodinâmica até 30 dias depois foram analisadas como desfechos, tendo o procedimento hemodinâmico como procedimento-índice. Além disso, os seguintes dados foram coletados: variáveis demográficas e clínicas; necessidade de novos procedimentos hemodinâmicos; atraso na realização dos procedimentos; tempos sintoma-porta, porta-balão e porta-mesa; e disponibilidade e uso adequado de equipamento de proteção individual (EPI).

O protocolo do estudo, o termo de consentimento informado e outros documentos pertinentes à pesquisa foram submetidos ao Comitê de Ética em Pesquisa do centro coordenador, enviados para avaliação e aprovados pela Comissão Nacional de Ética em Pesquisa (CONEP, protocolo CAAE 30564720.0.0000.5292).

### Análise estatística

Para a análise dos dados, foram utilizados modelos de regressão logística, tendo o primeiro modelo sido implementado com o uso de *elastic net* para seleção prévia de variáveis independentes. Outros modelos de regressão logística foram implementados e re-estimados por máxima verossimilhança, retendo as variáveis com significância estatística (Material Suplementar 2).

Técnicas de *machine learning* foram empregadas com os modelos não paramétricos de árvores de classificação, e o desfecho composto foi ‘óbito e complicações CV e não CV’.

A análise de correspondência simples foi inicialmente realizada usando-se uma tabela de contingência em cujas linhas estavam os eventos: complicações (CV e não CV), IM e ausência de eventos, e as combinações de eventos (IM & complicações; complicações & óbito; IM & complicações & óbito). As colunas continham as 37 variáveis restantes, representando as características clínicas e demográficas. Para reduzir o tamanho do conjunto de dados, realizou-se nova análise de correspondência, com as mesmas variáveis da análise inicial nas linhas da tabela de contingência. As colunas, no entanto, continham as 25 variáveis clínicas e demográficas que mais haviam contribuído nas duas primeiras dimensões. Os pontos de linha ou coluna (variáveis) com perfis similares encontram-se próximos. Nas linhas ou colunas, as variáveis correlacionadas negativamente situam-se em posições opostas em relação à origem. Quanto mais afastadas da origem, maior sua contribuição na dimensão.

Os dados relacionados a óbito foram analisados utilizando-se um modelo log-linear, tendo a dependência entre as variáveis discretas sido avaliada com o índice V de Cramer. A análise gráfica foi realizada com um gráfico representando as conexões obtidas a partir de um modelo log-linear. A espessura de cada aresta associada com as variáveis definidas pelos vértices é proporcional ao índice V de Cramer.

Os seguintes pacotes do software estatístico R foram usados na análise de dados: *partykit* , para as árvores de sobrevida; *ca* e *FactoMineR* , para a análise de correspondência; e *igraph* para os gráficos. O nível de significância de 5% foi adotado.^12 - 15^

## Resultados

Este estudo incluiu 1.282 pacientes, 435 dos quais (33,9%) apresentaram IM como se segue: 239 (54,9%) tinham IM com supra de ST (IMCSST) e 196 (45,1%), IM sem supra de ST (IMSSST). Dos 1.282 pacientes, 29 apresentaram complicações CV [parada cardiorrespiratória (2), IM periprocedural (2), nova revascularização (24), AVC (1)], 47 apresentaram complicações não CV [tamponamento cardíaco relacionado ao procedimento (4), sangramento procedural (15), sepse pulmonar (2), choque séptico (2), síndrome respiratória aguda (3), infecção respiratória (21)], e 31 morreram. Não houve diferença estatisticamente significativa entre os óbitos por causas CV e não CV (teste exato de Fisher: p = 0,3951).

O diagnóstico de COVID-19 foi confirmado em 77 pacientes (6%), com mortalidade de 15,58% e complicações não CV em 6,49%.

A Tabela 1 apresenta as principais características dos pacientes. A maioria era do sexo masculino (810, 63%), tinha escolaridade mínima de 9 anos e era atendida em unidades do Sistema Único de Saúde brasileiro (SUS), que é público e universal. Os pacientes tinham múltiplos fatores de risco CV, principalmente hipertensão arterial (943, 74%) e sedentarismo (1.050, 82%). Vale ressaltar que a maioria dos pacientes apresentava significativa doença arterial coronariana (DAC) (812, 63%), sendo o achado de um trombo intracoronariano mais frequente na presença de IMCSST (8, 3,4%) e COVID-19 (3, 4%). Um terço dos pacientes positivos para COVID-19 não tinha DAC significativa (obstrução maior do que 50%).

**Tabela 1:** – Características gerais dos pacientes submetidos a procedimentos de cardiologia intervencionista durante a pandemia de COVID-19

Variáveis	IMSSST (n=196) N (%)	IMCSST (n=239) N (%)	Todos os pacientes (n=1.282) N (%)	COVID (n=77) N (%)
**Características gerais**				
Idade (anos)	64,35	61,22	63,14	65,98
Homens	126 (64,28)	165 (69,04)	810 (63,18)	51 (72,86)
Escolaridade: Nenhuma	3 (1,53)	15 (6,28)	50 (3,90)	3 (3,90)
Até 9 anos	114 (58,16)	144 (60,25)	694 (54,13)	47 (61,04)
10 a 12 anos	48 (24,49)	51 (21,34)	299 (23,32)	17 (22,08)
Mais de 15 anos	31 (15,82)	29 (12,13)	239 (18,64)	10 (12,99)
Tipo de internação: SUS	108 (55,10)	176 (73,64)	726 (56,63)	50 (64,94)
Privada	9 (4,59)	5 (2,09)	127 (9,91)	1 (1,30)
Convênio	79 (40,31)	58 (24,27)	429 (33,46)	26 (33,77)
**História clínica**				
COVID	21 (10,71)	19 (7,95)	77 (6,01)	77 (100,00)
Hipertensão arterial	147 (75,00)	154 (64,44)	943 (73,56)	60 (77,92)
Diabetes	73 (37,24)	70 (29,29)	432 (33,70)	31 (40,26)
Tabagismo	33 (16,84)	65 (27,20)	189 (14,74)	8 (10,39)
Dislipidemia	79 (40,31)	76 (31,80)	628 (48,99)	29 (37,66)
Obesidade	48 (24,49)	55 (23,01)	319 (24,88)	20 (25,97)
Sedentarismo	161 (82,14)	203 (84,94)	1050 (81,90)	70 (90,91)
Doença renal prévia	20 (10,20)	2 (0,84)	58 (4,52)	10 (12,99)
IM prévio	34 (17,35)	29 (12,13)	223 (17,39)	19 (24,68)
AVC prévio	12 (6,12)	8 (3,35)	46 (3,59)	4 (5,19)
Insuficiência cardíaca	24 (12,24)	10 (4,18)	126 (9,83)	11 (14,29)
Doença pulmonar prévia	3 (1,53)	9 (3,77)	43 (3,35)	4 (5,19)
História familiar de DAC	68 (34,69)	87 (36,40)	567 (44,23)	25 (32,47)
Revascularização prévia (percutânea/cirúrgica)	36 (18,37)	25 (10,46)	259 (20,20)	18 (23,38)
Uso prévio de AAS	174 (88,78)	211 (88,28)	1018 (79,41)	64 (83,12)
Uso prévio de estatina	129 (65,82)	118 (49,37)	705 (54,99)	35 (45,45)
Uso prévio de betabloqueador	106 (54,08)	90 (37,66)	585 (45,63)	29 (37,66)
**Características dos procedimentos**				
DAC significativa	147 (75,00)	202 (84,52)	812 (63,34)	48 (62,34)
Trombo	3 (1,53)	8 (3,35)	17 (1,33)	3 (3,90)
Sem DAC significativa	35 (17,86)	21 (8,79)	386 (30,11)	21 (27,27)
Outros achados	11 (5,61)	8 (3,35)	67 (5,23)	5 (6,49)*
Artéria culpada: TCE	3 (1,53)	6 (2,51)	-	-
ADA	46 (23,47)	100 (41,84)	-	-
ACx	29 (14,80)	19 (7,95)	-	-
ACD	21 (10,71)	69 (28,87)	-	-
**Desfechos**				
Complicações cardiovasculares	6 (3,06)	5 (2,09)	29 (2,26)	2 (2,60)
Complicações não cardiovasculares	16 (8,16)	6 (2,51)	47 (3,67)	5 (6,49)
Óbito	7 (3,57)	9 (3,77)	31 (2,42)	12 (15,58)

IMCSST: infarto do miocárdio com supra de ST; IMSSST: infarto do miocárdio sem supra de ST; IM: infarto do miocárdio; DAC: doença arterial coronariana; AVC: acidente vascular cerebral; AAS: ácido acetilsalicílico; TCE: tronco de coronária esquerda; ADA: artéria descendente anterior; ACx: artéria circunflexa; ACD: artéria coronária direita. (*) outros achados: ponte intramiocárdica, dissecção de artéria coronária, dissecção de aorta e espasmo coronariano grave.

**Figura 1:**
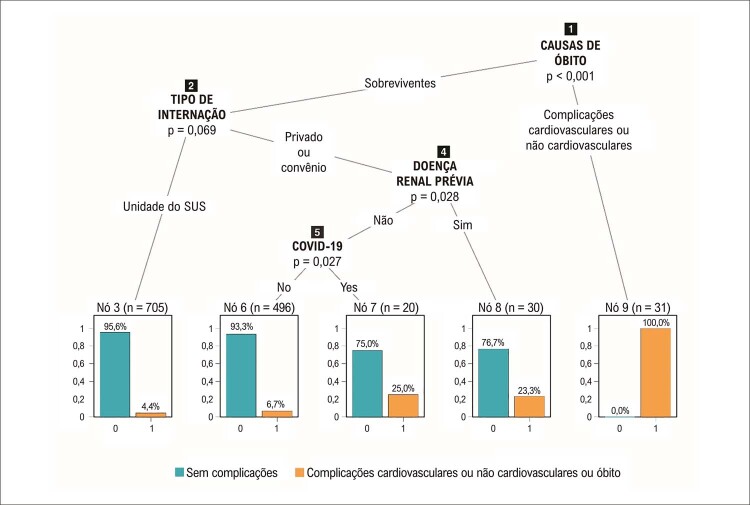
– Árvore de classificação para o desfecho composto ‘óbito & complicações cardiovasculares e não cardiovasculares’ em todos os pacientes do registro (n=1.282). O nó principal (nó 1) indica a ocorrência ou não de óbito. O nó 9 representa os 31 pacientes que morreram de complicações cardiovasculares e não cardiovasculares (COVID-19, sepse, causas respiratórias, etc). Dos sobreviventes, 705 foram atendidos em uma unidade do SUS, 31 dos quais tiveram complicações (nó 3). Dos 546 pacientes não atendidos em uma unidade do SUS (nó 4), 30 tinham insuficiência renal e 20 tinham COVID-19, 7 dos quais tiveram complicações cardiovasculares (nó 8) e 5 tiveram complicações não cardiovasculares (nó 7).

Todas as mortes (n=31; 2,5%) foram associadas com complicações. Dos 1.251 sobreviventes, 546 (43,6%) não foram atendidos em uma unidade do SUS (hospitais privados e hospitais conveniados). Desses 546 pacientes, 30 apresentaram insuficiência renal prévia, 7 dos quais (23%) tiveram complicações. Daqueles sem insuficiência renal prévia, 20 tiveram diagnóstico confirmado de COVID-19, 5 dos quais (25%) com complicações ( Figura 1 ).

**Figura 2:**
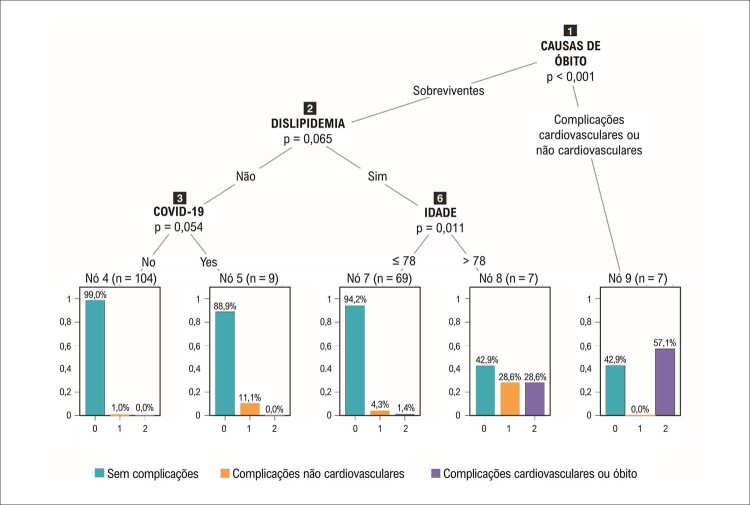
– Árvore de classificação para o desfecho ‘complicações (cardiovasculares e não cardiovasculares)’ em 196 pacientes com IMSSST. O nó principal (nó 1) indica a ocorrência ou não de óbito. O nó 9 representa os 7 pacientes que morreram, 3 dos quais por complicações cardiovasculares. Entre os sobreviventes, a presença de dislipidemia e idade superior a 78 anos (nó 8, n=7) associou-se a complicações não cardiovasculares (n=2) e cardiovasculares (n=2). Entre aqueles com idade ≤ 78 anos (nó 7, n=69), 3 pacientes tiveram complicações não cardiovasculares e 1 teve complicações cardiovasculares. Entre os sobreviventes sem dislipidemia, o diagnóstico de COVID-19 (nó 5, n=9) associou-se com complicações não cardiovasculares (n=1).

Dos 196 pacientes com IMSSST, 182 não tiveram complicação e 7 morreram [4 (57%) de complicações associadas à COVID-19 e 3 (43%) de complicações CV]. Para o desfecho composto (‘óbito & complicações CV e não CV’), um tempo porta-mesa superior a 12 horas foi associado a 30,8% de complicações, 25% das quais devidas à COVID-19. É crucial notar que, no início da pandemia, os atrasos foram atribuídos à COVID-19 apenas ou à dificuldade de acesso ao atendimento em saúde, que podiam ser responsabilizados pelas complicações isoladamente ou em combinação. Entre os sobreviventes, a presença de dislipidemia e idade superior a 78 anos associou-se a 28,6% das complicações CV (n=2) e a igual número de complicações não CV. Entre aqueles sem dislipidemia, foram identificados 9 pacientes com COVID-19, 1 dos quais (11,1%) apresentou complicações não CV ( Figura 2 ).

**Figura 3:**
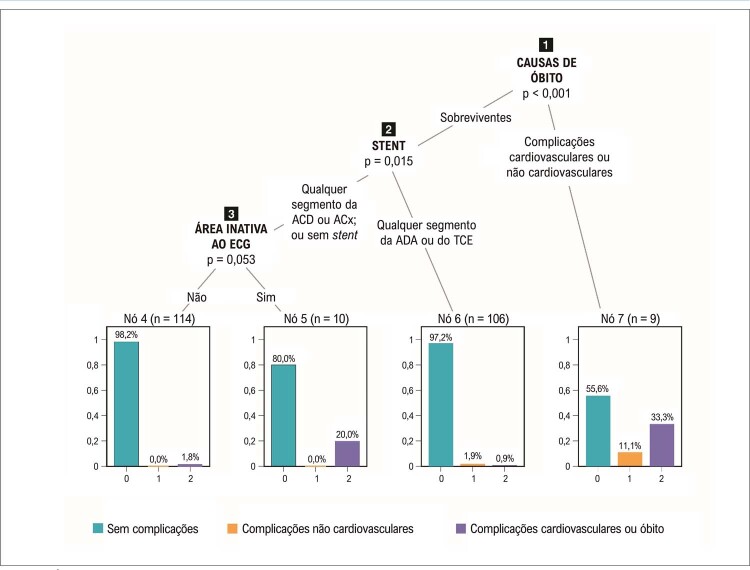
– Árvore de classificação para o desfecho ‘complicações (cardiovasculares e não cardiovasculares)’ em 239 pacientes com IMCSST. O nó principal (nó 1) indica a ocorrência ou não de óbito. Nove pacientes morreram, 3 com complicações cardiovasculares e 1 com complicações de COVID-19 (nó 7). Entre os sobreviventes, a presença de 1 stent no TCE ou na ADA (nó 6, n=106) foi associada com complicações não cardiovasculares (n=2) e cardiovasculares (n=1). Entre os sobreviventes que não precisaram de stent ou que receberam stent na ACD ou ACx, a presença de área inativa ao ECG (nó 5, n=10) foi associada com complicações cardiovasculares em 2 pacientes. Dois pacientes sem área inativa ao ECG (nó 4, n=114) apresentaram complicações cardiovasculares relacionadas ao procedimento. TCE: tronco de coronária esquerda; ADA: artéria descendente anterior; ACx: artéria circunflexa; ACD: artéria coronária direita.

Dos 239 pacientes com IMCSST submetidos a angioplastia primária, 19 tiveram diagnóstico confirmado de COVID-19, 228 não tiveram complicação, 3 tiveram complicações não CV e 9 morreram. Das 9 mortes, 3 (33,3%) foram associadas com complicações CV e 1 (11%) com complicações de COVID-19. Entre os sobreviventes, 103 pacientes (97,2%) apresentaram lesões de tronco de coronária esquerda e da artéria descendente anterior abordadas com sucesso, enquanto 20% daqueles com lesões de artéria coronária direita e circunflexa apresentaram complicações CV na presença de IM prévio. A abordagem de 112 (98%) pacientes que não apresentavam área inativa ao eletrocardiograma foi bem-sucedida ( Figura 3 ).

**Figura 4:**
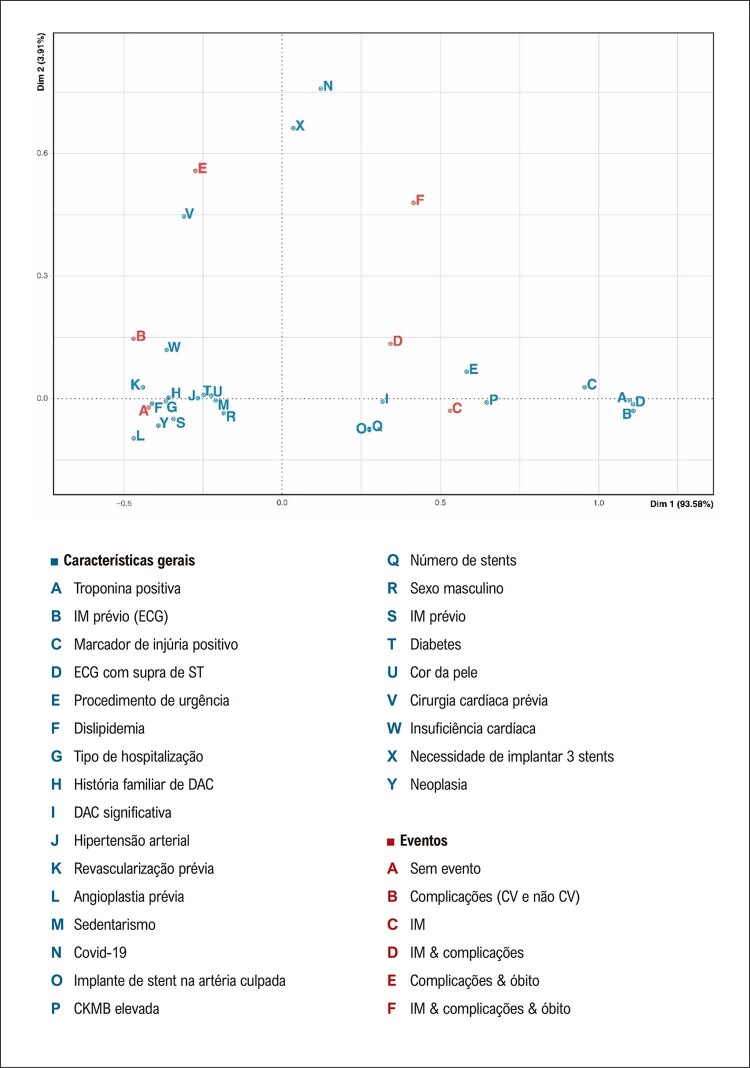
Análise de correspondência para os eventos: complicações (CV e não CV), IM, ausência de eventos e as combinações de eventos (‘IM & complicações’; ‘complicações & óbito’; ‘IM & complicações & óbito’). O diagnóstico de COVID-19 e a necessidade de implantar 3 stents associaram-se com ‘IM & complicações & óbito’. IMCSST, número de stents, implante de stent na artéria culpada e admissão de emergência no laboratório de hemodinâmica contribuíram para explicar a combinação de eventos ‘IM & complicações’ sem óbito. Para a combinação de eventos ‘complicações & óbito’, ‘cirurgia cardíaca prévia’ foi a variável identificada. O evento ‘complicações (CV e não CV)’ associou-se com insuficiência cardíaca, diabetes, revascularização prévia, IM prévio, história de DAC, sexo masculino, hipertensão arterial, sedentarismo e dislipidemia. Duas dimensões explicaram 97% da variabilidade dos dados. DAC: doença arterial coronariana; CV: cardiovascular; IM: infarto do miocárdio.

A Figura 4 apresenta o resultado da análise de correspondência simples, onde duas dimensões concentraram 97,5% da variabilidade dos dados. As características gerais que contribuíram para a combinação de eventos ‘IM & complicações & óbito’ foram a presença de COVID-19 e a necessidade de implante de três *stents* , provavelmente refletindo a carga aterosclerótica. Em relação à combinação de eventos ‘IM & complicações’ sem óbito, as seguintes características contribuíram: IMCSST, número de *stents, stents* alocados em artérias culpadas e necessidade de admissão de emergência no laboratório de hemodinâmica. Para a combinação de eventos ‘complicações & óbito’, cirurgia cardíaca prévia foi a característica identificada. Quanto à ocorrência de todas as complicações, tanto CV quanto não CV, as seguintes características contribuíram para explicar o evento: insuficiência cardíaca, diabetes, revascularização prévia, IM prévio, história de DAC, sexo masculino, hipertensão arterial, sedentarismo e dislipidemia.

**Figura 5:**
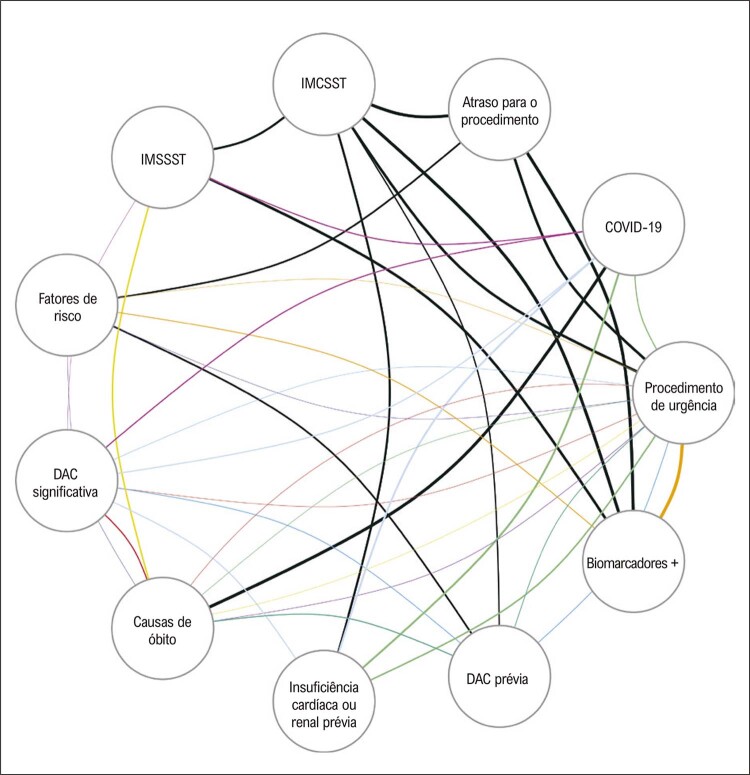
– Análise gráfica das conexões obtidas no modelo log-linear implementado para analisar as variáveis associadas com óbito. As interações de segunda ordem estão representadas em preto; as outras cores representam as interações de terceira ordem. A espessura das arestas é proporcional ao índice V de Cramer, que mede a dependência entre as variáveis discretas. IMCSST: infarto do miocárdio com supra de ST; IMSSST: infarto do miocárdio sem supra de ST; DAC: doença arterial coronariana.

A Figura 5 mostra um gráfico usando o índice V de Cramer calculado para pacientes que morreram, considerando 11 variáveis clínicas dicotômicas: IMSSST; IMCSST; COVID-19; causas de óbito; fatores de risco; DAC prévia; insuficiência cardíaca ou renal prévia; biomarcadores positivos; procedimento urgente; atraso para o procedimento; e DAC significativa. O modelo log-linear foi implementado com 11 variáveis e suas conexões representadas em um gráfico. Entre as interações de terceira ordem, as associações entre ‘fatores de risco’, ‘procedimento urgente’ e ‘biomarcadores positivos’ destacaram-se (índice V de Cramer = 0,6), como esperado. Entre as interações de segunda ordem, as seguintes associações sobressaíram: COVID-19, DAC significativa (MI ou revascularização prévia) e IMCSST; DAC significativa, procedimento urgente e complicações CV como causas de óbito; COVID-19, insuficiência cardíaca ou renal e procedimento urgente; COVID-19, insuficiência cardíaca ou renal e DAC prévia; COVID-19, insuficiência cardíaca ou renal e complicações CV como causas de óbito. Todas essas associações ressaltam a interação dos múltiplos fatores que contribuíram para a morte dos pacientes submetidos a procedimentos percutâneos de cardiologia intervencionista no início da pandemia de COVID-19.

Todos os laboratórios de hemodinâmica participantes deste estudo relataram disponibilidade e uso adequado de EPI padronizado durante os procedimentos.

## Discussão

O Brasil foi afetado pela COVID-19 de maneira desigual, o que impactou os desfechos dos procedimentos de cardiologia intervencionista realizados durante a pandemia. Dos 1.282 pacientes do registro RBCI-COVID19, 107 (8,4%) apresentaram complicações ou morreram e 77 (6%) apresentaram infecção concomitante pelo SARS-CoV-2. Nesse registro, a maioria dos procedimentos de hemodinâmica foi ressarcida pelo SUS e a presença de insuficiência renal e infecção pelo SARS-CoV-2 foi associada com o desfecho composto ‘óbito & complicações’ ( Tabela 1 ).

Um estudo observacional de séries temporais comparando procedimentos de cardiologia intervencionista registrados pelo SUS entre janeiro e maio de 2020 com os do mesmo período de 2016 e 2019, assim como com os valores projetados através de regressão linear para o ano de 2020, relatou uma redução de 27% naqueles procedimentos durante a pandemia de COVID-19.^11^ Além disso, aquele estudo relatou reduções de 9% e 5% nas mortes hospitalares e na taxa de letalidade intra-hospitalar por IM, respectivamente.^11^ Entretanto, aquele estudo não avaliou se houve aumento na mortalidade domiciliar nem mediu a influência da infecção concomitante pelo SARS-CoV-2 nas mortes e complicações relacionadas aos procedimentos de cardiologia intervencionista.

No presente estudo, dos pacientes diagnosticados com IMSSST, 3,6% morreram, a maioria (57%) com complicações da COVID-19. Além disso, um terço dos pacientes com IMSSST foi submetido ao cateterismo após pelo menos 12 horas do início dos sintomas e um quarto apresentou complicações da COVID-19. Daqueles com diagnóstico de IMCSST e submetidos a angioplastia primária, 3,8% morreram, um terço de complicações CV e o restante de complicações da COVID-19. Um estudo brasileiro com 152 pacientes consecutivos com COVID-19 suspeita ou confirmada e IM (IMSSST=69, IMCSST=83), submetidos a angiografia coronariana em 17 centros terciários, de 14 de abril a 28 de junho de 2020, relatou mortalidade hospitalar global de 23,7%. Os autores atribuíram a alta mortalidade hospitalar às morfologias coronarianas complexas, superposição de doença aterosclerótica e trombo, além de perfusão miocárdica deficiente (blush 0/1).^9^

Uma série de 18 casos de IMCSST e COVID-19 reportada no início da pandemia de COVID-19 mostrou taxa de mortalidade hospitalar de até 50-70%, tendo metade dos pacientes sido submetida a angiografia coronariana, dois terços dos quais apresentaram doença obstrutiva. Os autores levantaram a hipótese de que a lesão miocárdica dos pacientes com COVID-19 poderia ser devida a ruptura de placa aterosclerótica, tempestade de citocinas, espasmo coronariano, microtrombos ou lesão endotelial ou vascular direta.^16^ Rodríguez-Leor et al. compararam os resultados de 91 pacientes com COVID-19 aos de 919 pacientes sem COVID-19 tratados com angioplastia primária para IMCSST em um registro multicêntrico espanhol com 42 sítios. Os pacientes menos frequentemente receberam tratamento prévio com ácido acetilsalicílico ou um inibidor de P2Y12, mas mais frequentemente apresentaram insuficiência cardíaca, foram submetidos a procedimentos mecânicos de trombectomia e receberam inibidores de GP IIb/IIIa, sugerindo maior carga trombótica. Esses pacientes apresentaram uma incidência 2,6 vezes maior de choque cardiogênico após o procedimento e um aumento de 4 vezes na trombose aguda de *stent* com consequente mortalidade hospitalar 4 vezes maior (23,1%).^6^ Os achados desses dois registros foram semelhantes aos do RBCI-COVID19, com incidência bem menor de desfechos adversos, como complicações CV e morte.

O registro ISACS-STEMI COVID-19, com 6.609 pacientes com IMCSST submetidos a angioplastia primária em 77 centros europeus de 18 países em março/abril de 2019 e 2020, relatou uma redução de 19% no tratamento percutâneo de IM no início da pandemia em comparação a 2019 [(razão da taxa de incidência: 0,811 (IC 95%: 0,78 - 0,84; p < 0,0001)]. A mortalidade em 2020 foi maior do que em 2019 (192 mortes, 6,8% vs. 169 mortes, 4,9%, OR: 1,41; IC 95%: 1,15 - 1,71; p < 0,001), sendo que 18 dos 62 pacientes com COVID-19 morreram (29%). A heterogeneidade entre os centros não foi relacionada à incidência de óbito por COVID-19. Os autores relataram significativo aumento nos tempos porta-balão e de isquemia total, assim como significativa interação entre hipertensão arterial e mortalidade.^17^ Rodriguez-Leor et al., em um registro multicêntrico, retrospectivo e observacional de pacientes com IMCSST, mais de 94% dos quais tratados com angioplastia primária, em 75 centros na Espanha, relataram diminuição de 22,7% no número de pacientes com IMCSST em comparação ao mesmo período anterior à pandemia. Além disso, encontraram maior mortalidade hospitalar durante o surto de COVID-19 (7,5% vs. 5,1%) e incidência de 6,3% de infecção confirmada por SARS-CoV-2 durante a hospitalização.^18^ Em comparação a esses dois registros, o RBCI-COVID19 mostrou menor mortalidade (3,77%), que foi maior entre aqueles com infecção concomitante por SARS-CoV-2 (15,58%). Ademais, a análise de correspondência realizada no presente estudo mostrou associação entre hipertensão arterial e complicações totais ( Figura 4 ).

O registro prospectivo COVID-ACS compilou dados de 144 pacientes com IMCSST e 121 pacientes com síndrome coronariana aguda sem supra de ST (SCA-SSST) de 55 centros internacionais de 1 de março a 31 de julho de 2020. O registro mostrou que os tempos desde o início dos sintomas até a admissão foram significativamente prolongados e a mortalidade significativamente maior nos pacientes com COVID-IMCSST (22,9% vs. 5,7%; p < 0,001) e naqueles com COVID SCA-SSST (6,6% vs. 1,2%; p < 0,001), mesmo quando a análise de propensão foi ajustada para comorbidades (razão de chance do subgrupo IMCSST: 3,33 [IC 95%: 2,04 - 5,42]). Taxas excessivas e mortalidade por choque cardiogênico contribuíram significativamente para os piores desfechos nos pacientes com IMCSST e positivos para COVID-19.^19^

Um estudo avaliando um período pré-COVID-19 (1 de janeiro de 2019 a 14 de março de 2020) e um período pandêmico (15 de março a 4 de abril de 2020) em 51 hospitais do estado de Nova York certificados para realizar intervenção coronariana percutânea relatou uma redução de 43% nos procedimentos/semana nos hospitais de condados com alta densidade de COVID-19 e de apenas 4% naqueles de condados com baixa densidade da doença, sem diferença nas taxas de mortalidade hospitalar ajustadas para risco. Segundo os autores, tal redução deveu-se mais ao não comparecimento dos pacientes aos hospitais nas regiões de alta densidade de COVID-19 do que à tentativa de se evitar a intervenção coronariana percutânea.^20^ Por outro lado, em 83 pacientes com IMCSST e COVID-19, Popovic et al. relataram mortalidade hospitalar de 8,4%, IM trombótico com oclusão coronariana não aterosclerótica em 11 e maior embolização distal após o procedimento. Os autores correlacionaram os achados angiográficos e a mortalidade com a concomitância da infecção pelo SARS-CoV-2.^2^

De Luca et al., em um grande registro retrospectivo multicêntrico com 109 centros de alto volume de intervenção coronariana percutânea primária da Europa, América Latina (incluindo centros do Brasil), Sudeste Asiático e Norte da África, arrolando 16.674 pacientes com IMCSST submetidos a angioplastia primária entre março/junho de 2019 e 2020, observaram significativa redução nas angioplastias primárias no período estudado (razão da taxa de incidência: 0,843, IC 95%: 0,825 - 0,861, p<0,0001). Houve significativo aumento no tempo porta-balão [40 (25-70) min vs. 40 (25-64) min, p=0,01] e no tempo total de isquemia [225 (135–410) min vs. 196 (120-355) min, p <0,001], o que pode ter contribuído para elevar a mortalidade hospitalar (6,5% vs. 5,3%, p<0,001) e em 30 dias (8% vs. 6,5%, p=0,001) durante a pandemia. Além disso, naquele grande registro retrospectivo multicêntrico, as mortalidades hospitalares foram maiores do que as relatadas no RBCI-COVID19. Os autores daquele grande estudo sugeriram que a diferença nos resultados dos centros de intervenção possa ser atribuída às diferenças locais nas instituições de saúde e na conduta nas emergências CV devidas à COVID-19, com impacto tanto no receio de transmissão da doença quanto no risco de morte súbita fora do hospital.^21^

Gupta et al. relataram mortalidade hospitalar de 35% em um registro multicêntrico nos Estados Unidos, com 2.215 pacientes com COVID-19 internados em unidades de tratamento intensivo de 65 hospitais em todo o país, ressaltando a importância da intervenção precoce para tais pacientes. Os fatores associados a morte incluíram idade avançada, sexo masculino, obesidade, DAC, câncer e disfunção orgânica aguda, com grande variação entre os hospitais.^22^ Uma análise retrospectiva de 254.288 brasileiros com idade mínima de 20 anos, hospitalizados com COVID-19 confirmada e cadastrados no Sistema de Informação da Vigilância Epidemiológica da Gripe (SIVEP-Gripe) entre 16 de fevereiro e 15 de agosto de 2020 (semanas epidemiológica 8-33), relatou mortalidade hospitalar global de 38%. Os autores encontraram disparidades regionais no sistema de saúde e ressaltaram a necessidade de se melhorar o acesso ao cuidado de alta qualidade, particularmente nos países de renda baixa e média.^23^ Entretanto, vale ressaltar que a maioria dos pacientes que necessitam de cuidado CV para doenças cardíacas isquêmicas, vasculares periféricas ou cardíacas estruturais pode não estar infectada com o novo coronavírus. É necessário garantir que a população geral continue a se beneficiar dos cuidados CV, em especial aqueles relacionados às síndromes isquêmicas agudas.^23^

Um estudo recente que analisou a preparação para a pandemia e a COVID-19 em 177 países, de 1º de janeiro de 2020 a 30 de setembro de 2021, demonstrou que os fatores que explicaram a maior variação da taxa de letalidade relacionada à COVID-19 no mesmo período foram o perfil etário do país (46,7% [II_95 _18,4 - 67,6] de variação), o produto interno bruto per capita (3,1% [II_95_ 0,3 - 8,6] de variação) e o índice de massa corporal nacional médio (1,1% [II_95 _0,2 - 2,6] de variação). A maioria das variações entre países nas taxas de infecção cumulativas não pôde ser explicada. Os índices de preparação para pandemias, que visam medir a capacidade de segurança em saúde, não foram significativamente associados a taxas de infecção padronizadas nem a razões fatalidade-infecção. Os autores sugeriram que o aumento da promoção da saúde para riscos modificáveis estaria associado à redução de fatalidades nesse cenário.^24^

Dados os enormes desafios relacionados à interação entre envolvimento cardíaco na COVID-19 e doenças respiratórias e CV crônicas e agudas, estudos adicionais devem abordar a alta carga das doenças cardiopulmonares associadas com procedimentos CV, considerando o equilíbrio ótimo entre investigações custo-efetivas e benefício para os pacientes e prevenção de óbito e complicações ( Figura 5 ). Além disso, há necessidade de se minimizar as desigualdades na prestação de cuidados em saúde e maximizar o suporte social, pois o envolvimento cardíaco na COVID-19 continua a ser uma importante questão de saúde pública.^25^

Com base nesses achados, podemos supor que, em países continentais como o Brasil, coexista uma multiplicidade de fatores relacionados com a letalidade dos procedimentos cardiovasculares de alta complexidade. A presença de atendimento de saúde universalizado e centrado nos Programas de Saúde da Família pode ter contribuído para as menores taxas de letalidade observadas no registro RBCI-COVID19. Novos estudos que analisem as características individuais de cada país precisarão modelar as variáveis explicativas das variações nas taxas de infecção cumulativas envolvendo as DCV que têm poder incremental para aumentar a letalidade pela COVID-19, especialmente nas faixas etárias mais avançadas e nos indivíduos com índice de massa corporal elevado ( Figura Central ).

**Figura Central:**
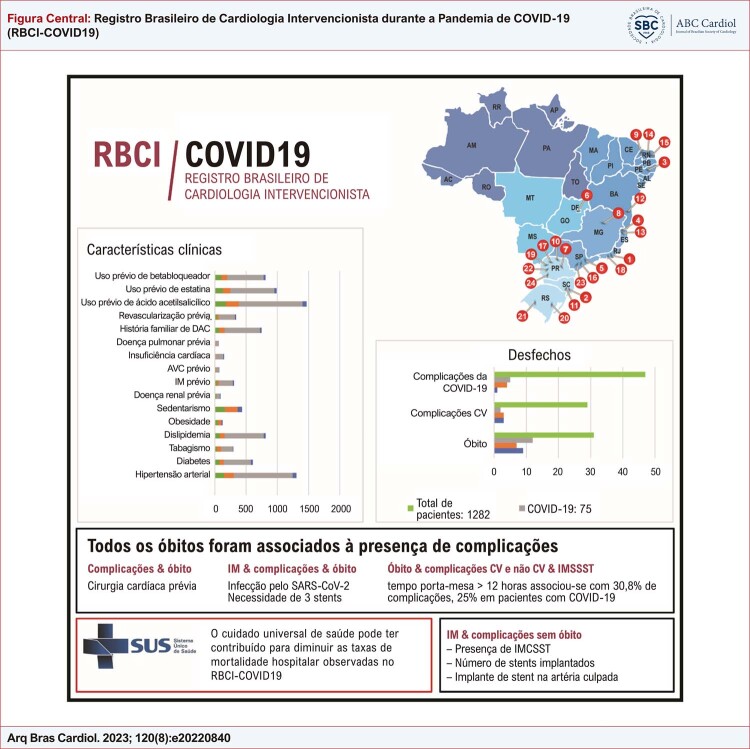
: Registro Brasileiro de Cardiologia Intervencionista durante a Pandemia de COVID-19 (RBCI-COVID19)

AVC: acidente vascular cerebral; CV: cardiovascular; DAC: doença arterial coronariana; IM: infarto do miocárdio; IMCSST: infarto do miocárdio com supra de ST.

Uma limitação do presente estudo foi a coleta de dados, que se mostrou desafiadora durante a pandemia devido a dados ausentes ou de qualidade potencialmente baixa, especialmente quanto à descrição detalhada dos procedimentos de cardiologia intervencionista. Entretanto, este é o maior registro brasileiro de cardiologia intervencionista que demonstrou a importância do atendimento no SUS com reduzida mortalidade hospitalar de pacientes com IMCSST e IMSSST. Vale ressaltar que a mortalidade foi influenciada pela concomitância da COVID-19, com um aumento de 5 vezes em comparação à de pacientes sem COVID-19 ( Figura Central ). Uma força deste estudo foi a análise de dados com o uso de inteligência artificial, que permitiu a identificação dos padrões dos pacientes com complicações e óbito no período.

## Conclusão

Todos os óbitos foram associados com a presença de complicações. Quanto ao desfecho composto (‘óbito & complicações CV e não CV’) nos pacientes com IMSSST, um tempo porta-mesa superior a 12 horas foi associado com 30,8% de complicações, 25% das quais ocorreram na presença de COVID-19. As variáveis que contribuíram para o desfecho ‘IM & complicações & óbito’ foram a presença de infecção por SARS-CoV-2 e a necessidade de implante de três *stents* . Com relação ao desfecho ‘IM & complicações’ sem óbito, a presença de IMCSST, o número de *stents* e o implante de *stent* na artéria culpada contribuíram para explicar aquele desfecho. Quanto ao desfecho ‘complicações & óbito’, cirurgia cardíaca prévia foi a variável identificada como contribuindo para o desfecho. Vale notar que a COVID-19 teve influência na morte e nas complicações não fatais de pacientes submetidos a procedimentos de cardiologia intervencionista durante a pandemia.

### Perspectivas

- O que já se sabe?

Durante 2020, a pandemia impediu que os pacientes buscassem tratamento para suas DCV, em especial as agudas, como IM, com consequente atraso nas admissões hospitalares.

- O que é novo?

A inteligência artificial mostrou uma multiplicidade de fatores relacionados com a letalidade dos procedimentos cardiovasculares de alta complexidade. A presença de atendimento de saúde universalizado pode ter contribuído para reduzir as taxas de mortalidade hospitalar observadas no registro RBCI-COVID19.

- O que está por vir?

Novos estudos que analisem as características individuais de cada país precisarão modelar as variáveis explicativas das variações nas taxas de infecção cumulativas envolvendo as DCV e letalidade por COVID-19.

### Lista de investigadores

Viviana Guzzo Lemke, Maria Sanali Souza Paiva, Giordana Zeferino Mariano, Thales Siqueira Alves. Esmeralci Ferreira, Leonardo Avany Nunes, Flávio Roberto Azevedo de Oliveira, Rodrigo Cantarelli, Renato Giestas Serpa, Breno de Siqueira, Luciano de Moura Santos, Stefan Silveira, Frederico Toledo Campo Dall`Orto, Wangles Jotão Geraldo, Cesar Dusilek, Marcelo Harada Ribeiro, Thais Chang Valente Tamazato, Felipe Bortot Cesar, André Francisco de Paula Antonangelo, Luís Fernando Alves Campos, Ricardo Barbosa, Luiz Gustavo Pauletti, Maria Cristina Meira Ferreira, Eder Voltolini, Tiago Vendruscolo, Alessandra Teixeira de Oliveira, Alysson Moço Faidiga, Fernanda Marinho Mangione, Marcel Rogers Ravanelli, Emilia Matos do Nascimento, Glaucia Maria Moraes de Oliveira
