# Supplementary material for: Registro Brasileiro de Cardiologia Intervencionista durante a Pandemia de COVID-19 (RBCI-COVID19)
Source: Arq Bras Cardiol. 2023 Aug 10;120(8):e20220840. [Article in Portuguese] doi: 10.36660/abc.20220840 (PMC10519241; doi:10.36660/abc.20220840)
Supplement: Translation

# Translation: Brazilian Registry of Interventional Cardiology during the COVID-19 Pandemic (RBCI-COVID19)

**Authors:** Viviana Guzzo Lemke, Maria Sanali Souza Paiva, Giordana Zeferino Mariano, Thales Siqueira Alves, Esmeralci Ferreira, Leonardo Avany Nunes, Flavio Roberto Azevedo Oliveira, Rodrigo Cantarelli, Emilia Matos do Nascimento, Gláucia Maria Moraes de Oliveira

**Affiliations:** 1 Brasil Cardiocare, Curitiba, PR – Brasil; 2 Brasil Hospital Universitário Onofre Lopes – HUOL, Natal, RN – Brasil; 3 Brasil Hospital São João Batista, Criciúma, SC – Brasil; 4 Brasil Hospital Universitário Pedro Ernesto, Rio de Janeiro, RJ – Brasil; 5 Brasil Universidade do Estado do Rio de Janeiro – Doenças do Tórax, Rio de Janeiro, RJ – Brasil; 6 Brasil Hospital Dom Helder Câmara, Recife, PE – Brasil; 7 Brasil Universidade do Estado do Rio de Janeiro, Rio de Janeito, RJ – Brasil; 8 Brasil Universidade Federal do Rio de Janeiro, Rio de Janeiro, RJ – Brasil

**Keywords:** COVID-19, Myocardial Infarction, Percutaneous Coronary Intervention, Coronary Angiography, Coronary Artery Disease

## Abstract

**Background:**

At the beginning of the COVID-19 pandemic, patients with myocardial infarction (MI) took longer to present to hospitals because of fear of contamination and health care access difficulties.

**Objectives:**

To assess interventional cardiology procedures performed during the COVID-19 pandemic and its implications for MI approach.

**Methods:**

Prospective registry of 24 cardiac catheterization laboratories in Brazil, with adult patients undergoing interventional cardiology procedures between May 26 and November 30, 2020. The outcomes were cardiovascular (CV) and non-CV complications, death, and MI. Concomitant COVID-19 was confirmed using RT-PCR. Machine learning techniques were used with nonparametric Classification Trees models, and Simple Correspondence Analysis, with R statistical software package. Significance level adopted of 5%.

**Results:**

This study included 1282 patients, 435 of whom (33.9%) had MI as follows: ST-segment elevation MI (STEMI), 239 (54.9%); and non-ST-segment elevation MI (NSTEMI), 196 (45.1%). Of the 1282 patients, 29 had CV complications, 47 had non-CV complications, and 31 died. The diagnosis of COVID-19 was confirmed in 77 patients (6%), with 15.58% mortality and non-CV complications in 6.49%. Most patients had significant coronary artery disease (63%), and an intracoronary thrombus was more often found in the presence of STEMI (3.4%) and COVID-19 (4%). A door-to-table time longer than 12 hours in NSTEMI was associated with 30.8% of complications, 25% in COVID-19 patients.

**Conclusions:**

All deaths were preceded by CV or non-CV complications. The presence of COVID-19 was associated with death and non-fatal complications of patients undergoing interventional cardiology procedures during the pandemic.

## Introduction

Up to the beginning of 2022, around 23 million COVID-19 cases and 621 000 deaths due to the disease had been registered in Brazil.^1^During 2020, the pandemic kept patients from seeking treatment for their cardiovascular diseases (CVD), especially the acute ones, such as myocardial infarction (MI) and stroke, with a consequent decline in and/or delay to hospital admissions because hospitals were perceived as dangerous places regarding the risk of infection.^2 - 4^

A study conducted in Brazil, using the public database from the Civil Registration System, has assessed total and cardiovascular (CV) excess deaths occurring in the epidemiological weeks 12-22 of 2020 in six Brazilian cities with the highest numbers of deaths from COVID-19 (São Paulo, Rio de Janeiro, Fortaleza, Recife, Belém, and Manaus). These numbers were compared with those of that same period in 2019. There was a decrease in the excess of specified CV deaths (MI and stroke) in parallel with an increase in unspecified CV and household deaths.^5^

Several studies have reported deaths and significant cardiac complications, such as MI, thrombotic events, heart failure, myopericarditis, and cardiac arrhythmias, in COVID-19 patients with or without previous CVD.^2 - 6^The damage of COVID-19 to the CV system is probably multifactorial and can result initially from an imbalance between high metabolic demand and low cardiac reserve associated with systemic inflammation and thrombogenesis, in addition to SARS-CoV-2 direct cardiac damage.^7 , 8^

A few studies have assessed the impact of the pandemic on interventional cardiology procedures and the pandemic implications for the invasive approach to MI in the Brazilian population.^9 , 10^Therefore, this study aimed to analyze percutaneous CV procedures performed during the COVID-19 pandemic, as well as its implications for the invasive approach to MI, using a multicenter registry of cardiac catheterization laboratories (cath labs) in Brazil (RBCI-COVID19).

## Methods

This is a multicenter observational registry compiling data from all consecutive patients aged at least 18 years, both sexes, undergoing a CV procedure at 24 cardiac cath labs in Brazil from May 26 to November 30, 2020 (Supplemental Material 1). The exclusion criteria were as follows: refusal to provide written informed consent, admission for neurological or peripheral vascular procedures, or implantation of artificial cardiac stimulation devices.

All cardiac cath labs that volunteered to adhere to the study after the registry was announced were included. Data were collected from standardized electronic case report forms, available online, and inserted by the cath lab teams trained by the coordinating center.^11^

The outcomes observed were death from any cause, CV complications (cardiac tamponade, cardiorespiratory arrest, new revascularization, stroke, pulmonary embolism, acute heart failure, cardiogenic shock) and non-CV complications (respiratory infection, sepsis, septic shock, procedural bleeding), and MI. The complications occurring from arrival to the cath lab (interventional cardiology procedure as an index procedure) up to 30 days after that were analyzed and considered outcomes. In addition, the following data were collected: demographic and clinical variables; need for new interventional cardiology procedures; delay to procedure; symptom-to-admission, door-to-balloon, and door-to-table times; and individual protection equipment (IPE) availability and proper use.

The study protocol, the informed consent form, and other documents pertinent to this research were submitted to the coordinating center’s Research Ethics Committee, sent for evaluation to and approved by the National Research Ethics Committee (protocol CAAE 30564720.0.0000.5292).

### Statistical analysis

Data were analyzed by use of logistic regression models, and the first model was implemented by using elastic net for the previous selection of independent variables. Other logistic regression models were implemented and re-estimated by use of maximum likelihood, retaining the variables with statistical significance (Supplemental Material 2).

Machine learning techniques were used with nonparametric models of classification trees, and the composite outcome was ‘death & CV and non-CV complications’.

Simple correspondence analysis was initially conducted using a contingency table considering in the rows of events: complications (CV and non-CV), MI, no event, and combinations of events (MI & complications; complications & death; and MI & complications & death). The columns contained the remaining 37 variables, representing clinical and demographic characteristics. To reduce the size of data set, a new correspondence analysis was conducted, having the same variables of the initial analysis in the rows of the contingency table. The columns, however, comprised the 25 clinical and demographic variables that contributed most to the first two dimensions. Row or column (variables) points with similar profiles are found close to each other. In the rows or columns, variables negatively correlated are located opposite in relation to the origin. The farther they are from the origin, the greater their contribution to the dimension.

Death data were analyzed using a log-linear model, and Cramer’s V index assessed the dependence between discrete variables. The graphical analysis was conducted using a graph representing the connections obtained from the log-linear model. The thickness of each edge associated with the variables defined by the vertices is proportional to the Cramer’s V index.

Data were analyzed by using the following R statistical software package: partykit for the survival trees; *ca* and *FactoMineR* for the correspondence analysis; and igraph for the graphs. A 5% significance level was adopted.^12 - 15^

## Results

This study included 1282 patients, 435 of whom (33.9%) had MI as follows: 239 (54.9%) with ST-segment elevation MI (STEMI) and 196 (45.1%) with non-ST-segment elevation MI (NSTEMI). Of the 1282 patients, 29 had CV complications [cardiorespiratory arrest (2), periprocedural MI (2), new revascularization (24), stroke (1)], 47 had non-CV complications [procedure-related cardiac tamponade (4), procedural bleeding (15), pulmonary sepsis (2), septic shock (2), acute respiratory syndrome (3), respiratory infection (21)], and 31 died. There was no statistically significant difference between deaths from CV causes and non-CV causes (Fisher’s exact test: p = 0.3951)

The diagnosis of COVID-19 was confirmed in 77 patients (6%), with mortality of 15.58% and non-CV complications in 6.49%.

The patients’ major characteristics are described in Table 1 . Most patients were of the male sex (810, 63%), had a minimum schooling of 9 years, and were cared for at units of the Brazilian Unified Public Health System (SUS), which is public and universal. The patients had multiple CV risk factors, mainly arterial hypertension (943, 74%) and sedentary lifestyle (1050, 82%). It is worth noting that most patients had significant coronary artery disease (CAD) (812, 63%), and an intracoronary thrombus was more often found in the presence of STEMI (8, 3.4%) and COVID-19 (3, 4%). One third of the COVID-19-positive patients had no significant CAD (lumen obstruction greater than 50%).

**Table 1:** – General characteristics of patients undergoing interventional cardiology procedures during the COVID-19 pandemic

Variables	NSTEMI (n=196) N (%)	STEMI (n=239) N (%)	All patients (n=1282) N (%)	COVID (n=77) N (%)
**General characteristics**				
Age (years)	64.35	61.22	63.14	65.98
Men	126 (64.28)	165 (69.04)	810 (63.18)	51 (72.86)
Schooling: None	3 (1.53)	15 (6.28)	50 (3.90)	3 (3.90)
Up to 9 years	114 (58.16)	144 (60.25)	694 (54.13)	47 (61.04)
10 to 12 years	48 (24.49)	51 (21.34)	299 (23.32)	17 (22.08)
Over 15 years	31 (15.82)	29 (12.13)	239 (18.64)	10 (12.99)
Type of hospitalization: SUS	108 (55.10)	176 (73.64)	726 (56.63)	50 (64.94)
Private	9 (4.59)	5 (2.09)	127 (9.91)	1 (1.30)
Health insurance	79 (40.31)	58 (24.27)	429 (33.46)	26 (33.77)
**Clinical history**				
COVID	21 (10.71)	19 (7.95)	77 (6.01)	77 (100.00)
Arterial hypertension	147 (75.00)	154 (64.44)	943 (73.56)	60 (77.92)
Diabetes	73 (37.24)	70 (29.29)	432 (33.70)	31 (40.26)
Smoking	33 (16.84)	65 (27.20)	189 (14.74)	8 (10.39)
Dyslipidemia	79 (40.31)	76 (31.80)	628 (48.99)	29 (37.66)
Obesity	48 (24.49)	55 (23.01)	319 (24.88)	20 (25.97)
Sedentary lifestyle	161 (82.14)	203 (84.94)	1050 (81.90)	70 (90.91)
Previous kidney disease	20 (10.20)	2 (0.84)	58 (4.52)	10 (12.99)
Previous MI	34 (17.35)	29 (12.13)	223 (17.39)	19 (24.68)
Previous stroke	12 (6.12)	8 (3.35)	46 (3.59)	4 (5.19)
Heart failure	24 (12.24)	10 (4.18)	126 (9.83)	11 (14.29)
Previous pulmonary disease	3 (1.53)	9 (3.77)	43 (3.35)	4 (5.19)
Family history of CAD	68 (34.69)	87 (36.40)	567 (44.23)	25 (32.47)
Previous revascularization (percutaneous/surgical)	36 (18.37)	25 (10.46)	259 (20.20)	18 (23.38)
Previous use of acetylsalicylic acid	174 (88.78)	211 (88.28)	1018 (79.41)	64 (83.12)
Previous use of statin	129 (65.82)	118 (49.37)	705 (54.99)	35 (45.45)
Previous use of beta-blocker	106 (54.08)	90 (37.66)	585 (45.63)	29 (37.66)
**Characteristics of the procedures**				
Significant CAD	147 (75.00)	202 (84.52)	812 (63.34)	48 (62.34)
Thrombus	3 (1.53)	8 (3.35)	17 (1.33)	3 (3.90)
No significant CAD	35 (17.86)	21 (8.79)	386 (30.11)	21 (27.27)
Other findings	11 (5.61)	8 (3.35)	67 (5.23)	5 (6.49)*
Culprit artery: LMCA	3 (1.53)	6 (2.51)	-	-
ADA	46 (23.47)	100 (41.84)	-	-
CxA	29 (14.80)	19 (7.95)	-	-
RCA	21 (10.71)	69 (28.87)	-	-
**Outcomes**				
Cardiovascular complications	6 (3.06)	5 (2.09)	29 (2.26)	2 (2.60)
Non-cardiovascular complications	16 (8.16)	6 (2.51)	47 (3.67)	5 (6.49)
Death	7 (3.57)	9 (3.77)	31 (2.42)	12 (15.58)

STEMI: ST-elevation myocardial infarction; NSTEMI: non-ST-elevation myocardial infarction; MI: myocardial infarction; CAD: coronary artery disease; LMCA: left main coronary artery; ADA: anterior descending artery; CxA: circumflex artery; RCA: right coronary artery. (*) other findings: intramyocardial bridge, coronary artery dissection, aortic dissection, and severe coronary spasm.

All deaths (n=31; 2.5%) were associated with complications. Of the 1251 survivors, 546 (43.6%) were not cared for at a SUS unit (private and health insurance hospitals). Of those 546 patients, 30 had previous kidney failure, 7 of whom (23%) had complications. Of those without previous kidney failure, 20 had a confirmed COVID-19 diagnosis, 5 of whom (25%) had complications ( Figure 1 ).

**Figure 1:** – Classification tree for the composite outcome ‘death & cardiovascular and non-cardiovascular complications’ in all patients of the registry (n=1282). The major node (node 1) indicates the occurrence or not of death. Node 9 represents the 31 patients who died from cardiovascular and non-cardiovascular complications (COVID-19, sepsis, respiratory causes, etc). Of the survivors, 705 were cared for at a SUS unit, 31 of whom had complications (node 3). Of the 546 patients not cared for at a SUS unit (node 4), 30 had kidney failure and 20 had COVID-19, 7 of whom had cardiovascular complications (node 8) and 5 had non-cardiovascular complications (node 7).

Of the 196 patients with NSTEMI, 182 had no complication and 7 died [4 (57%) from complications associated with COVID-19 and 3 (43%) from CV complications]. For the composite outcome (death & CV and non-CV complications) a door-to-table time longer than 12 hours was associated with 30.8% of complications, 25% of which due to COVID-19. It is crucial to note that during the early days of the pandemic, the delays to procedures were attributed to COVID-19 alone or to difficulties in health care access, which could be responsible for the complications alone or together. Among the survivors, the presence of dyslipidemia and age over 78 years associated with 28.6% of CV complications (n=2) and an equal number of non-CV complications. Among those without dyslipidemia, 9 patients with COVID-19 were identified, 1 of whom (11.1%) had non-CV complications ( Figure 2 ).

**Figure 2:** – Classification tree for the outcome ‘complications (cardiovascular and non-cardiovascular)’ in 196 patients with NSTEMI. The major node (node 1) indicates the occurrence or not of death. Seven patients died, 3 of whom from cardiovascular complications (node 9). Among the survivors, the presence of dyslipidemia and age over 78 years (node 8, n=7) associated with non-cardiovascular (n=2) and cardiovascular (n=2) complications. Among those aged 78 years or under (node 7, n=69), 3 patients had non-cardiovascular complications and 1 had cardiovascular complications. Among survivors without dyslipidemia, the diagnosis of COVID-19 (node 5, n=9) associated with non-cardiovascular complications (n=1).

Of the 239 patients with STEMI who underwent primary angioplasty, 19 had a confirmed COVID-19 diagnosis, 228 had no complication, 3 had non-CV complications, and 9 died. Of the 9 deaths, 3 (33.3%) were associated with CV complications and 1 (11%) with complications from COVID-19. Among the survivors, 103 patients (97.2%) had their left main coronary artery and anterior descending artery lesions successfully approached, while 20% of those with right coronary artery and circumflex artery lesions had CV complications in the presence of previous MI. The 112 (98%) patients with no inactive area identified on the electrocardiogram were successfully approached ( Figure 3 ).

**Figure 3:** – Classification tree for the outcome ‘complications (cardiovascular and non-cardiovascular)’ in 239 patients with STEMI. The major node (node 1) indicates the occurrence or not of death. Nine patients died, 3 with cardiovascular complications and 1 with COVID-19 complications (node 7). Among the survivors, the presence of a stent in the LMCA or ADA (node 6, n=106) was associated with non-cardiovascular (n=2) and cardiovascular (n=1) complications. Among the survivors not requiring a stent or who underwent RCA or CxA stenting, the presence of an inactive area on ECG (node 5, n=10) was associated with cardiovascular complications in 2 patients. In the absence of an inactive area on ECG (node 4, n=114), 2 patients had cardiovascular complications related to the procedure. Note: RCA: right coronary artery; CxA: circumflex artery; ADA: anterior descending artery; LMCA: left main coronary artery.

Figure 4 shows the result of the simple correspondence analysis, evidencing that two dimensions retained 97.5% of data variation. The general characteristics contributing to the combination of events ‘MI & complications & death’ were the presence of COVID-19 and need for implantation of three stents, probably reflecting the atherosclerotic burden. Regarding the combination of events ‘MI & complications’ without death, the following characteristics contributed to explain that event: STEMI, number of stents, stenting of the culprit artery, and need for emergency admission to cath lab. For the combination of events ‘complications & death’, previous cardiac surgery was the characteristic identified. Regarding the occurrence of all complications, either CV or non-CV, the following characteristics contributed to explain that event: heart failure, diabetes, previous revascularization, previous MI, history of CAD, male sex, arterial hypertension, sedentary lifestyle, and dyslipidemia.

**Figure 4:** – Correspondence analysis for the events: complications (cardiovascular and non-cardiovascular), MI, absence of events, and the combinations of events: ‘MI & complications’; ‘complications & death’; and ‘MI & complications & death’. The diagnosis of COVID-19 and the need to implant three stents associated with ‘MI & complications & death’. STEMI, number of stents, stenting of the culprit artery, and emergency admission to the cath lab contributed to explain the combination of events ‘MI & complications’ without death. For the combination of events ‘complications & death’, ‘previous cardiac surgery’ was the variable identified. Complications (cardiovascular and non-cardiovascular) associated with heart failure, diabetes, previous revascularization, previous MI, history of CAD, male sex, arterial hypertension, sedentary lifestyle, and dyslipidemia. Two dimensions explained 97% of data variation. CAD: coronary artery disease; cath lab: catheterization laboratory; CV: cardiovascular; MI: myocardial infarction.

Figure 5 shows a graph using the Cramer’s V index calculated for patients who died, considering 11 dichotomized clinical variables: NSTEMI; STEMI; COVID-19; cause of death; risk factors; previous CAD; previous heart or kidney failure; positive biomarkers; urgent procedure; delay to procedure; and significant CAD. The log-linear model was implemented with 11 variables and their connections were represented by a graph. Of the third-order interactions, the links between ‘risk factors’, ‘urgent procedure’, and ‘positive biomarkers’ stood out (Cramer’d V index = 0.6), as expected. Of the second-order interactions, the following associations stood out: COVID-19, significant CAD (previous MI or revascularization), and STEMI; significant CAD, urgent procedure, and CV complications as causes of death; COVID-19, heart or kidney failure, and urgent procedure; COVID-19, heart or kidney failure, and previous CAD; COVID-19, heart or kidney failure, and CV complications as causes of death. All these associations highlight the interaction of multiple factors that contributed to the death of patients submitted to percutaneous procedures in the beginning of the COVID-19 pandemic.

**Figure 5:** – Graphical analysis representing the connections obtained in the log-linear model implemented to analyze the variables associated with death. Second-order interactions were characterized by the color black; the other colors represent the third-order interactions. Edge thicknesses are proportional to the Cramer’s V index, a measure of dependence between discrete variables. STEMI: ST-segment elevation myocardial infarction; NSTEMI: non-ST-segment elevation myocardial infarction; CAD: coronary artery disease.

All cardiac cath labs participating in this study reported standardized IPE availability and proper use during the procedures.

## Discussion

Brazil has been struck by COVID-19 in a heterogeneous manner, which has impacted on the outcomes of the interventional cardiology procedures performed during the pandemic. Of the 1282 patients in the RBCI-COVID19 registry, 107 (8.4%) either died or had complications, and 77 (6%) had concomitant SARS-CoV-2 infection. In that registry, most interventional cardiology procedures were reimbursed by the SUS, and the presence of kidney failure and SARS-CoV-2 infection was associated with the composite outcome ‘death & complications’ ( Table 1 ).

An observational time-series study comparing cardiac catheterizations registered by the SUS between January and May 2020 with those in the same period of 2016 and 2019, as well as with the values projected via linear regression methods for the year 2020, has reported a 27% reduction in those procedures during the COVID-19 pandemic.^11^That study has reported 9% and 5% reductions in in-hospital death and in-hospital mortality from MI, respectively.^11^However, that study neither assessed whether there was an increase in household mortality, nor measured the influence of concomitant SARS-CoV-2 infection on the deaths and complications related to cardiac catheterization procedures.

In the present study, of the patients diagnosed with NSTEMI, 3.6% died, most of whom (57%) with complications from COVID-19. In addition, one third of the patients with NSTEMI underwent catheterization 12 hours or more from symptom onset, and one fourth of them had COVID-19 complications. Of the patients diagnosed with STEMI and undergoing primary angioplasty, 3.8% died, one third from CV complications and the rest from COVID-19 complications. A Brazilian study with 152 consecutive patients with suspected or confirmed COVID-19 and MI (NSTEMI=69, STEMI=83), who underwent coronary angiography in 17 tertiary centers, from April 14 to June 28, 2020, has reported overall in-hospital mortality of 23.7%. The authors attributed the high in-hospital mortality to complex coronary morphologies, superposition of atherosclerotic disease and a thrombus, and deficient myocardial perfusion (blush 0/1).^9^

A case series of 18 patients with STEMI and COVID-19 reported at the beginning of the COVID-19 pandemic has shown in-hospital mortality rate of up to 50-70%, and half of the patients underwent coronary angiography, two-thirds of whom had obstructive disease. The authors hypothesized that the COVID-19 myocardial damage could be due to rupture of an atherosclerotic plaque, cytokine storm, coronary spasm, microthrombi, or direct endothelial or vascular damage.^16^Rodriguez-Leor et al. have reported the results of 91 patients with COVID-19 as compared to those of 919 patients without COVID-19 treated with primary angioplasty for STEMI in a Spanish multicenter registry with 42 sites. The patients were less often pretreated with acetylsalicylic acid or P2Y12 inhibitor, more often had heart failure, underwent mechanical thrombectomy procedures, and received GP IIb/IIIa inhibitors, suggesting a higher thrombotic burden. Those patients had a 2.6-fold greater incidence of postprocedural cardiogenic shock and a 4-fold increase in acute stent thrombosis with consequent 4-fold higher in-hospital mortality (23.1%).^6^The findings of those two registries were similar to the ones observed in the RBCI-COVID19 registry, with a much lower incidence of adverse outcomes, such as CV complications and death.

The ISACS-STEMI COVID-19 registry, with 6609 patients with STEMI undergoing primary angioplasty in 77 European centers from 18 countries in March/April 2019 and 2020, has reported a 19% reduction in the percutaneous treatment of MI at the beginning of the pandemic as compared to 2019 [(incidence rate ratio: 0.811 (95% CI: 0.78 to 0.84; p < 0.0001)]. Mortality in 2020 was higher than that in 2019 (192 deaths, 6.8% vs. 169 deaths, 4.9%, OR: 1.41; 95% CI: 1.15 to 1.71; p < 0.001), and 18 of the 62 patients with COVID-19 died (29%). The heterogeneity between centers was not related to the incidence of death due to COVID-19. The authors reported a significant increase in door-to-balloon and total ischemia times, as well as a significant interaction between arterial hypertension and mortality.^17^Rodriguez-Leor et al., in a multicenter, retrospective, observational registry of patients with STEMI, more than 94% treated with primary angioplasty, in 75 centers in Spain, have reported a 22.7% decrease in the number of patients with STEMI as compared to the same period before the pandemic. In addition, they found a higher in-hospital mortality during the COVID-19 outbreak (7.5% vs 5.1%), and a 6.3% incidence of confirmed SARS-CoV-2 infection during hospitalization.^18^As compared to those two registries, the RBCI-COVID19 registry showed lower mortality (3.77%), which was higher among those with concomitant SARS-CoV-2 infection (15.58%). In addition, the correspondence analysis conducted in the present study showed association between arterial hypertension and total complications ( Figure 4 ).

A prospective COVID-ACS registry compiled data of 144 patients with STEMI and 121 patients with non-ST-segment elevation acute coronary syndrome (NSTE-ACS) from 55 international centers from March 1 to July 31, 2020. It showed that the symptom-to-admission times were significantly prolonged and mortality significantly higher in COVID-STEMI (22.9% vs. 5.7%; p < 0.001) and in COVID NSTE-ACS (6.6% vs. 1.2%; p < 0.001) patients, even when the propensity analysis was adjusted for comorbidities (STEMI subgroup odds ratio: 3.33 [95% CI: 2.04 to 5.42]). Excessive rates of and mortality from cardiogenic shock were major contributors to the worse outcomes in COVID-19-positive STEMI patients.^19^

A study assessing the pre-COVID-19 period (January 1, 2019, to March 14, 2020) and the COVID-19 period (March 15, 2020, to April 4, 2020) in 51 hospitals of the New York State certified to perform percutaneous coronary intervention has reported a decrease of 43% in procedures/week in the hospitals in high-density COVID-19 counties and of only 4% in the hospitals in low-density counties, with no difference in risk-adjusted in-hospital mortality rates. The authors related that decrease to patients not presenting to hospitals in high-density COVID-19 regions, rather than percutaneous coronary intervention being avoided.^20^On the other hand, in 83 patients with STEMI and COVID-19, Popovic B et al. have reported in-hospital mortality of 8.4%, thrombotic MI with non-atherosclerotic coronary occlusion in 11, and higher postprocedural distal embolization. Those authors correlated the angiographic findings and mortality with concomitance of SARS-CoV-2 infection.^2^

De Luca G et al., in a large multicenter retrospective registry in 109 high-volume primary percutaneous coronary intervention centers from Europe, Latin America (including centers from Brazil), South-East Asia, and North Africa, enrolling 16 674 patients with STEMI undergoing primary angioplasty from March/June 2019 to 2020, have observed a significant reduction in primary angioplasties in the period studied (incidence rate ratio: 0.843, 95% CI: 0.825 to 0.861, p<0.0001). There was a significant increase in the door-to-balloon time [40 (25-70) min vs. 40 (25-64) min, p=0.01] and in the total ischemia time [225 (135–410) min vs. 196 (120-355) min, p <0.001], which may have contributed to the increased in-hospital (6.5% vs. 5.3%, p<0.001) and 30-day (8% vs. 6.5%, p=0.001) mortalities during the pandemic. In addition, in that large multicenter retrospective registry, the in-hospital mortalities were higher than those reported in the RBCI-COVID19 registry. The authors of that large study have suggested that the difference in the results from the intervention centers might be attributed to the local differences in the health organizations and the management of the CV emergencies due to COVID-19, with impact on both the fear of disease transmission and the risk of sudden death outside the hospital.^21^

Gupta et al. have reported an in-hospital mortality of 35% in a multicenter registry in the United States, with 2215 patients with COVID-19 admitted to intensive care units at 65 hospitals across the country, emphasizing the importance of early intervention for those patients. The factors associated with death included older age, male sex, obesity, CAD, cancer, and acute organ dysfunction, with large inter-hospital variation.^22^A retrospective analysis of 254 288 Brazilians aged 20 years or older hospitalized with confirmed COVID-19 and registered in the Brazilian Influenza Epidemiological Surveillance Information System (SIVEP-Gripe), between February 16 and August 15, 2020 (epidemiological weeks 8-33), has reported overall in-hospital mortality of 38%. The authors have found regional disparities within the health system and emphasized the need to improve the access to high-quality care particularly in low- and middle-income countries.^23^However, it is worth noting that most patients requiring CV care for ischemic heart diseases, peripheral vascular diseases or structural heart diseases may not be infected with the new coronavirus. The general population needs to continue to benefit from CV care, especially that related to acute ischemic syndromes.^23^

A recent study assessing pandemic preparedness and COVID-19 in 177 countries, from January 1, 2020 to September 30, 2021, has evidenced the following factors explaining the higher variation in COVID-19 fatality rate in the same period: population’s age distribution (46.7% [II_95_18.4–67.6] of the variation), gross domestic product per capita (3.1% [II_95_0.3–8.6] of the variation), and mean national body mass index (1.1% [II_95_0.2–2.6] of the variation). Most variations in cumulative infection rates across countries could not be explained. The pandemic preparedness indices, which aim to measure health security capacity, were not significantly associated with standardized infection rates nor infection-fatality ratios. The authors have suggested that increasing health promotion for modifiable risks would be associated with a reduction of fatalities in this scenario.^24^

Given the enormous challenges related to the interaction between cardiac involvement in COVID-19 and chronic and acute respiratory and CV diseases, further studies must address the high burden of cardiopulmonary diseases associated with CV procedures considering the optimal balance between cost-effective investigations and benefit to patients and prevention of death and complications ( Figure 5 ). Furthermore, minimizing the inequalities in healthcare provision and maximizing social support are necessary because cardiac involvement in COVID-19 continues to be a significant public health issue.^25^

Considering those findings, we can assume that in countries of continental dimensions, such as Brazil, there are multiple factors related to the lethality of high-complexity CV procedures. The presence of universal health care centered on Family Health Programs might have contributed to lower the in-hospital mortality rates observed in the RBCI-COVID19 registry. Further studies analyzing the individual characteristics of each country need to model the explanatory variables of variations in the cumulative infection rates involving CVD with incremental power to increase COVID-19 lethality, especially in more advanced age ranges and individuals with high body mass index ( Central Illustration ).

**Central Illustration:** : Brazilian Registry of Interventional Cardiology during the COVID-19 Pandemic (RBCI-COVID19)

CAD: coronary artery disease; MI: myocardial infarction; CV: cardiovascular; STEMI: ST-segment elevation myocardial infarction.

A limitation of the present study was data collection, which proved to be a challenge during the pandemic because of missing and potentially low-quality data, especially regarding the detailed description of cardiac catheterization procedures. However, this is the largest Brazilian registry of interventional cardiology that showed the importance of the SUS care with reduced in-hospital mortality in STEMI and NSTEMI patients. It is worth noting that mortality was influenced by the concomitance of COVID-19, with a 5-fold increase as compared to that of patients without COVID-19 ( Central Illustration ). One strength of this study was data analysis by use of artificial intelligence, which enabled the identification of the patterns of the patients with complications and death in the period.

## Conclusion

All deaths were associated with the presence of complications. Regarding the composite outcome (death & CV and non-CV complications) in patients with NSTEMI, a door-to-table time longer than 12 hours was associated with 30.8% of the complications, 25% of which occurred in the presence of COVID-19. The variables contributing to the outcome ‘MI & complications & death’ were the presence of SARS-CoV-2 infection and the need for implantation of three stents. Regarding the outcome ‘MI & complications’ without death, the presence of STEMI, the number of stents, and stenting of the culprit artery contributed to explain that outcome. Regarding the outcome ‘complications and death’, ‘previous cardiac surgery’ was the variable identified as contributing to that outcome. It is worth noting that COVID-19 influenced the death and non-fatal complications of patients undergoing interventional cardiology procedures during the pandemic.

### Perspectives

- What is known?

During 2020, the pandemic kept patients from seeking treatment for their CVDs, especially the acute ones, such as MI, with a consequent delay to hospital admissions.

- What is new?

Artificial intelligence showed a multiplicity of factors related to the lethality of high-complexity CV procedures. The presence of universal health care might have contributed to lower the in-hospital mortality rates observed in the RBCI-COVID19 registry.

- What is next?

Further studies analyzing the individual characteristics of each country need to model the explanatory variables of variations in the cumulative infection rates involving CVD and COVID-19 lethality.

### Investigators list

Viviana Guzzo Lemke, Maria Sanali Souza Paiva, Giordana Zeferino Mariano, Thales Siqueira Alves. Esmeralci Ferreira, Leonardo Avany Nunes, Flávio Roberto Azevedo de Oliveira, Rodrigo Cantarelli, Renato Giestas Serpa, Breno de Siqueira, Luciano de Moura Santos, Stefan Silveira, Frederico Toledo Campo Dall`Orto, Wangles Jotão Geraldo, Cesar Dusilek, Marcelo Harada Ribeiro, Thais Chang Valente Tamazato, Felipe Bortot Cesar, André Francisco de Paula Antonangelo, Luís Fernando Alves Campos, Ricardo Barbosa, Luiz Gustavo Pauletti, Maria Cristina Meira Ferreira, Eder Voltolini, Tiago Vendruscolo, Alessandra Teixeira de Oliveira, Alysson Moço Faidiga, Fernanda Marinho Mangione, Marcel Rogers Ravanelli, Emilia Matos do Nascimento, Glaucia Maria Moraes de Oliveira
